# Zeaxanthin-independent energy quenching and alternative electron sinks cause a decoupling of the relationship between the photochemical reflectance index (PRI) and photosynthesis in an evergreen conifer during spring

**DOI:** 10.1093/jxb/erv427

**Published:** 2015-09-18

**Authors:** Emmanuelle Fréchette, Christopher Y. S. Wong, Laura Verena Junker, Christine Yao-Yun Chang, Ingo Ensminger

**Affiliations:** ^1^Department of Biology, University of Toronto at Mississauga, 3359 Mississauga Road, ON, Canada; ^2^Graduate Department of Cell & Systems Biology, University of Toronto, Toronto, ON, Canada; ^3^Graduate Department of Ecology and Evolutionary Biology, University of Toronto, Toronto, ON, Canada

**Keywords:** Climate change, Eastern white pine (*Pinus strobus*), leaf pigments, light-use efficiency of photosynthesis, photochemical and non-photochemical quenching, photochemical reflectance index, spring recovery of photosynthesis, xanthophyll cycle.

## Abstract

In evergreen conifers, the winter down-regulation of photosynthesis and its recovery during spring are the result of a reorganization of the chloroplast and adjustments of energy-quenching mechanisms. These phenological changes may remain undetected by remote sensing, as conifers retain green foliage during periods of photosynthetic down-regulation. The aim was to assess if the timing of the spring recovery of photosynthesis and energy-quenching characteristics are accurately monitored by the photochemical reflectance index (PRI) in the evergreen conifer *Pinus strobus*. The recovery of photosynthesis was studied using chlorophyll fluorescence, leaf gas exchange, leaf spectral reflectance, and photosynthetic pigment measurements. To assess if climate change might affect the recovery of photosynthesis, seedlings were exposed to cold spring conditions or warm spring conditions with elevated temperature. An early spring decoupling of the relationship between photosynthesis and PRI in both treatments was observed. This was caused by differences between the timing of the recovery of photosynthesis and the timing of carotenoid and chlorophyll pool size adjustments which are the main factors controlling PRI during spring. It was also demonstrated that zeaxanthin-independent NPQ mechanisms undetected by PRI further contributed to the early spring decoupling of the PRI–LUE relationship. An important mechanism undetected by PRI seems to involve increased electron transport around photosystem I, which was a significant energy sink during the entire spring transition, particularly in needles exposed to a combination of high light and cold temperatures.

## Highlight

Decoupling of the photochemical reflectance index and photosynthesis in evergreen conifers during spring is caused by energy-quenching mechanisms that remain undetected by leaf reflectance measurements and remote sensing.

## Introduction

Recent increases in global temperature are causing large-scale changes in carbon cycling of the conifer-dominated northern forest ecosystems (Piao *et al.*, 2008; Zhang *et al.*, 2013; IPCC, 2014). Since photosynthesis is highly variable in space and time, its assessment with high spatial and temporal resolution is essential accurately to assess the actual and projected effects of global climate change on the carbon budget of these forests. Remote sensing can provide data across a range of spatial scales and has become an important source of information for modelling global carbon cycling. In particular, the photochemical reflectance index (PRI), a parameter that can be derived from remotely sensed spectral reflectance data, has recently received considerable attention for its ability to detect changes in the photosynthetic efficiency of leaves, canopies, and entire ecosystems (Garbulsky *et al.*, 2011).

The biological basis of PRI is its capacity to detect the spectral signature of pigments involved in the dissipation of excess light and, hence, the efficiency of photosynthesis. To ensure optimum plant performance in ever-changing light environments, leaves constantly acclimate to balance the amount of light absorbed and their requirements for energy utilization (Ensminger *et al.*, 2006). While it is advantageous to maximize the absorption of light energy to fuel photosynthesis, the absorption of excess light that exceeds the capacity of photosynthesis can produce harmful reactive oxygen species (Barber and Andersson, 1992; Hüner *et al.*, 1998; Niyogi, 2000). Plants use different strategies to maintain a balance between the energy they absorb and the energy utilized for photochemistry. One strategy is to regulate light absorption capacity by, for example, chloroplast relocation (Kodama *et al.*, 2008). Another strategy is the regulation of light-use efficiency (LUE), i.e. the partitioning of absorbed light energy between photochemical and non-photochemical pathways (Demmig-Adams and Adams, 2006; Ensminger *et al.*, 2006). Evergreen conifers retain most of their chlorophyll content throughout the year and adjust LUE by various mechanisms in response to environmental conditions and depending on the season. During summer, the harmless removal of excess light, referred to as non-photochemical quenching (NPQ), is mediated by xanthophyll cycle pigments. Excess light energy rapidly induces the de-epoxidation of xanthophyll cycle pigments, i.e. the conversion of violaxanthin into the energy-quenching antheraxanthin and zeaxanthin (Niyogi *et al.*, 2005; Demmig-Adams and Adams, 2006; Verhoeven, 2014). Changes in the de-epoxidation status of xanthophyll cycle pigments (DEPS) affect leaf spectral reflectance at 531nm. This change in reflectance amplitude at 531nm is used to derive PRI by comparing it to the xanthophyll-insensitive band at 570nm (Gamon *et al.*, 1992, 1997; Peñuelas *et al.*, 1995). During winter, however, the xanthophyll cycle is arrested in a state primed for sustained energy quenching where zeaxanthin is retained, chlorophylls are partially degraded, photosystem (PS) II core units are reorganized (Ensminger *et al.*, 2006; Demmig-Adams and Adams, 2006; Verhoeven, 2014) and photoprotective pigments such as lutein and β-carotene are up-regulated (Adams and Demmig-Adams, 1994; Ottander *et al.*, 1995; Filella *et al.*, 2009; Verhoeven, 2014). These long-term, slowly reversible pigment adjustments are associated with this sustained mode of NPQ (Demmig-Adams and Adams, 2006).

Both short- and long-term pigment adjustments lead to changes in leaf spectral properties. These changes can be detected through leaf spectral reflectance measurements and have been used to measure PRI. In evergreen conifers, PRI was reported to be a good proxy for xanthophyll cycle dynamics over the course of a day (Nakaji *et al.*, 2006; Harris *et al.*, 2014), although there are reports that PRI has limitations to reflect the diurnal variation of xanthophyll de-epoxidation under excessive light (Kováč *et al.*, 2013). Recent studies have also reported good correlations between PRI and LUE over longer timescales, e.g. seasons (Garbulsky *et al.* 2011). It was suggested that PRI varies not only as a consequence of dynamic changes in xanthophyll pigments, but also as a result of long-term adjustments of pigment pools that allow plants to acclimate to changing environmental conditions throughout the year (Sims and Gamon, 2002; Garrity *et al.*, 2011; Wong and Gamon, 2015*a*, *b*; Hmimina *et al.*, 2015). Besides the adjustment of pigment pools, it is also important to note that, on the canopy scale, changes in leaf area, foliage clumping, and the distribution of shadow fraction affect the PRI signal that is detected from canopy spectral measurements (Hilker *et al.*, 2008, 2010). Long-term adjustments of chlorophyll and carotenoid pool sizes can be observed during spring in conifers, when photosynthesis recovers from winter stress (Ensminger *et al.*, 2004, 2008). These adjustments were shown to be a considerable source of PRI variation during the spring transition (Busch *et al.*, 2009; Porcar-Castell *et al.*, 2012; Wong and Gamon, 2015*a*, *b*). Over the course of the year, Wong and Gamon (2015*a*) observed that PRI variation in lodgepole and ponderosa pine (*Pinus contorta* D. and *P. ponderosa*) correlated better with the ratio of carotenoids per chlorophyll than xanthophyll cycle activity. In boreal Scots pine (*P. sylvestris* L.), Porcar-Castell *et al.* (2012) came to the same conclusion and reported that PRI was a good proxy for LUE during most of the year except during severe cold stress in the early spring. Similarly, Busch *et al.* (2009) observed that PRI could not explain variations in the quantum yield of PSII of Jack pine (*P. banksiana* Lamb.) seedlings during spring, when excess energy was predominantly dissipated via sustained quenching. The inability of the PRI to accurately detect changes in LUE during the winter–spring transition reflects the reorganization of the chloroplast during spring, that involves replacement of sustained NPQ by the rapidly reversible, flexible energy dissipation via the xanthophyll cycle (Demmig-Adams and Adams, 2006; Zarter *et al.*, 2006; Ensminger *et al.*, 2008).

In addition to sustained quenching, several zeaxanthin-independent NPQ mechanisms and alternative electron sinks might contribute to a discrepancy in the PRI–LUE relationship in conifers throughout the year (Busch *et al.*, 2009; Porcar-Castell *et al.*, 2012), although there is no direct evidence yet. NPQ mechanisms might include PSII and PSI reaction centre quenching (Öquist and Hüner, 2003), quenching of singlet excited chlorophyll by carotenoid pigments (Trebst, 2003; Telfer, 2005; Jahns and Holzwarth, 2012), and quenching via the lutein epoxide cycle (Matsubara *et al.*, 2012). Alternative electron sinks can include plastid terminal oxidase (PTOX)-mediated electron transport to oxygen (Savitch *et al.*, 2010) or photorespiration (Takahashi and Badger, 2011). Cyclic electron transport around PSI can also contribute significantly to the removal of excess energy during winter and early spring (Ivanov *et al.*, 2002). Recently it was suggested that the provision of ATP produced from cyclic electron transport maintains chloroplast integrity during chilling stress (Huang *et al.*, 2010) and supports the recovery from chilling stress. Furthermore, it has been established that the stoichiometry of the two photosystems adjusts to balance electron transport under cold conditions (Ivanov *et al.*, 2001; Ensminger *et al.*, 2004). Because some of these processes have the potential to adjust LUE downstream of PSII, they will remain undetected by PRI. Assessing the contribution of zeaxanthin-independent mechanisms to NPQ during the spring recovery of photosynthesis can, therefore, reveal sources of the decoupling observed in the PRI–LUE relationship during that season (Busch *et al.*, 2009; Porcar-Castell *et al.*, 2012; Wong and Gamon, 2015*b*).

In the boreal zone, the spring recovery of photosynthesis is largely driven by air temperature, which determines the physiological state of the chloroplast (Ensminger *et al.*, 2004). This includes the excitation pressure on PSII and PSI, energy partitioning between the photosystems, and the requirement for excess energy dissipation. The phenology of these events and, in turn, the PRI–LUE relationship, are likely to change as spring temperatures increase in the future (IPCC, 2014). For instance, the effects of warmer spring conditions on the transition from sustained NPQ to energy-dependent NPQ are uncertain, but will most likely affect the PRI–LUE relationship during that transitory period.

In the present study, the photosynthetic recovery of winter-acclimated Eastern white pine (*Pinus strobus* L.) seedlings exposed to cold or warm simulated spring conditions in controlled growth environments was followed in order (i) to investigate the effect of spring temperature on the transition from the sustained quenching mode of NPQ to energy-dependent quenching in conifer needles; (ii) to determine whether the spring timing of seasonal carotenoid and chlorophyll pool size adjustments, and thus of PRI recovery, are consistent with the recovery of LUE; and (iii) to identify zeaxanthin-independent NPQ mechanisms that contribute to the decoupling of the PRI–LUE relationship during the winter–spring transition.

## Materials and methods

### Plant material and growth conditions

Three-year-old Eastern white pine (*P. strobus* L.) seedlings were obtained in April 2011 and 2013 from a local nursery (Somerville Seedlings, Everett, Ontario, Canada), planted in a mixture of sand and sphagnum peat moss (1:3 v/v) and fertilized with 28:10:10 mineral fertilizer (Miracle-Gro, Scotts, Marysville, OH, USA). Seedlings were kept outside in an experimental garden at the University of Toronto at Mississauga (ON, Canada) until transfer to environmental growth chambers (Biochambers, Winnipeg, Canada) during December. The seedlings were acclimated for 6 weeks to simulated winter conditions (2/–5 °C day/night; 8h photoperiod at 400 μmol quanta m^–2^s^–1^). Winter-acclimated seedlings (Wi) were then shifted for 36 d to either a cold spring (Sp_C_) or a warm spring (Sp_W_) treatment. The temperature in Sp_C_ was set to 10/5 °C (day/night) and in Sp_W_ it was set to 15/10 °C (day/night) and the photoperiod was 12h in both spring treatments (Table 1). The light intensity was constantly monitored with a PAR sensor mounted at the top of the seedling canopy and maintained at an intensity of 1,400 μmol quanta m^–2^s^–1^ (Table 1). Incident sunlight under fluctuating natural conditions may reach light intensities well above 1,800 μmol quanta m^–2^s^–1^. However, preliminary experiments showed that a constant light intensity higher than 1,500 μmol quanta m^–2^s^–1^ over the full course of the photoperiod caused severe photodamage in our seedlings and light intensity in our growth chambers was therefore set to values not exceeding 1,400 μmol quanta m^–2^s^–1^. Wi seedlings were sampled and measured 1 d prior to transfer to spring conditions (day 0). Subsequent sampling and measurements of spring plants was done after transfer to spring conditions on days 1, 3, 6, 12, 18, 24, and 36 of the experiment. Measurements and samples of summer-acclimated needles were obtained from summer seedlings (Su) that had been kept outdoors in the experimental garden and were then acclimated for 6 weeks to simulated summer conditions (22/15 °C day/night; 14h photoperiod at 1,400 μmol quanta m^–2^s^–1^ light intensity). All measurements and needle sampling were performed on previous year needles of the topmost portion of the leader shoot. All data were obtained from two independent experiments performed in 2012 and 2014 using identical settings and protocols. In order to minimize any chamber effects further, the seedlings were rotated between chambers every 2 weeks.

**Table 1.** Overview of growth conditions within the chambers during the spring simulation

Experimental treatment	Air temperature	Photoperiod	Light intensity
	(°C; day/night)	(h)	(μmol m^–2^s^–1^)
Wi, winter	2/–5	8	400
Sp_C_, cold spring	10/5	12	1,400
Sp_W_, warm spring	15/10	12	1,400
Su, summer	22/15	14	1,400

### Chlorophyll fluorescence measurements

At each time point, chlorophyll-fluorescence measurements were performed using a Dual-PAM-100 (Walz, Effeltrich, Germany). Measurements were done on bundles of 10–15 needles that were aligned in parallel to form a single layer of needles in the leaf clip holder of the Dual-PAM-100. A saturating light pulse (SP) was applied to dark-adapted (pre-dawn) needles for the determination of *F*_o_ and *F*_m_ (minimal and maximum fluorescence). Maximum quantum yield of PSII (*F*_v_/*F*_m_) was calculated according to Genty *et al.* (1989):

$$\frac{F_{\text{v}}}{F_{\text{m}}}=\left(\frac{F_{\text{m}}-F_{\text{o}}}{F_{\text{m}}}\right)$$ (1)

The needles were then exposed to a sequence of 2.5-min intervals with actinic light of increasing intensity (0–2,000 μmol quanta m^–2^s^–1^), each step followed by a 400ms saturating pulse (SP) of 10,000 μmol quanta m^–2^s^–1^ for the determination of $F_{\text{m}}^{\prime }$ (maximum fluorescence of light-adapted needles), and a weak pulse of far-red light for determination of $F_{\text{o}}^{\prime }$ (minimal fluorescence of light-adapted needles). Energy partitioning parameters were calculated according to Hendrickson *et al.* (2004). The effective quantum yield of PSII of light-adapted needles (Φ_PSII_) reflects the proportion of light absorbed by PSII which is used for photochemistry and was calculated as:

$$\Phi {\;}_{\text{PSII}}=1-\frac{F_{\text{s}}}{F_{\text{m}}^{\prime }}$$ (2)

where *F*_s_ is the yield of fluorescence of a light-adapted sample. The proportion of light that is absorbed by PSII antenna and thermally dissipated via xanthophyll-regulated NPQ (Φ_NPQ_) was calculated as:

$$\Phi_{\text{NPQ}}=\frac{F_{\text{s}}}{F_{\text{m}}^{\prime }}-\frac{F_{\text{s}}}{F_{\text{m}}}$$ (3)

The energy quenched by fluorescence and dissipated constitutively (Φ_f,D_) was calculated as:

$$\Phi_{\text{f},\text{D}}=\frac{F_{\text{s}}}{F_{\text{m}}}$$ (4)

The electron transport rate of PSII (ETR_II_, in μmol electron m^–2^s^–1^) was calculated according to Genty *et al.* (1989):

$$ETR_{\text{II}}=\Phi_{\text{PSII}}\times PPFD\times \alpha \times d_{\text{II}}$$ (5)

where PPFD is the applied light intensity (μmol quanta m^–2^s^–1^), α is the absorptance, i.e. the fraction of incident light absorbed by leaves, and *d*_II_ the fraction of light directed to PSII. Values of α were calculated as α=1–transmittance–reflectance. Given the thickness of pine needles, transmittance was assumed to be 0. However, it should be noted that a small proportion of light can be transmitted through conifer needles (Lukeš *et al.*, 2013). Reflectance was measured with a Unispec-SC spectrometer over the 400–700nm wavelength range (Huang *et al.*, 2012). Values of *d*_II_ were calculated using the ratio of Φ_II_:Φ_I_ (see below) at low light intensity (60 μmol quanta m^–2^s^–1^), where CET is assumed to be absent and ETR_II_=ETR_I_ (Huang *et al.*, 2012).

The excitation pressure of PSII (1-qP) was calculated as:

$$1-qP=1-\frac{F_{\text{m}}^{\prime }-F_{\text{s}}}{F_{\text{m}}^{\prime }-F_{\text{o}}}$$ (6)

To assess energy partitioning characteristics at a diurnal time-scale, light response curves were measured on day 0 as well as on day 12 of the experiment. A dark-adapted bundle of needles was exposed to 10-min steps of increasing actinic light intensity (0–2,000 μmol quanta m^–2^s^–1^). At each light step, Φ_PSII_, Φ_NPQ_, and Φ_f,D_ were recorded.

### PSI absorbance measurements

Absorbance changes of the reaction centre chlorophyll of PSI (P700) were assessed simultaneously with chlorophyll fluorescence measurements using a Dual-PAM-100. The P700 signal (*P*) was calculated as the difference between the 875nm and 830nm transmittance signals. Firstly, P700 oxidation was transiently induced by applying a SP after far-red pre-illumination of dark-adapted needles. Briefly, after the SP, the minimal P700 signal was measured to capture a state of full P700 reduction. The difference between the fully reduced and fully oxidized states is denoted *P*_m_. Secondly, actinic illumination was applied with the same actinic light intensities and SPs used for fluorescence. Upon application of each maximum change of the P700 signal ($P_{\text{m}}^{\prime }$ ) was determined. Each SP was followed by a 1 s dark interval for the full reduction of P700 and determination of the minimal P700 signal (*P*_o_).

The three types of quantum yields of energy conversion in PSI were assessed according to Klughammer and Schreiber (1994) and calculated according to Pfündel *et al.* (2008). The effective quantum yield of PSI (Φ_PSI_) in the light was calculated as:

$$\Phi_{\text{PSI}}=\frac{P_{\text{m}}^{\prime }-P}{P_{\text{m}}}$$ (7)

The fraction of overall P700 that is oxidized in a given state due to a lack of electron donors (donor side limitation; Φ_ND_), was calculated as:

$$\Phi_{\text{ND}}=\frac{P-P_{\text{o}}}{P_{\text{m}}}$$ (8)

The fraction of overall P700 that cannot be oxidized by a saturation pulse in a given state due to a lack of electron acceptors (acceptor side limitation, Φ_NA_), was calculated as:

$$\Phi_{\text{NA}}=\frac{P_{\text{m}}-P_{\text{m}}^{\prime }}{P_{\text{m}}}$$ (9)

Analogous to ETR_II_, the electron transport rate of PSI (ETR_I_, in μmol electron m^–2^s^–1^) was calculated as:

$$ETR_{\text{I}}=\Phi_{\text{PSI}}\times PPFD\times \alpha \times d_{\text{I}}$$ (10)

where *d*_I_ was calculated as *d*_I_=1–*d*_II_ (Huang *et al.*, 2012)_._. Cyclic electron transport (CET, in μmol electron m^–2^s^–1^) was calculated according to Huang *et al.* (2012) as:

$$CET=ETR_{\text{I}}-ETR_{\text{II}}$$ (11)

### Photosynthetic gas exchange measurements

To assess variations in photosynthetic activity at a seasonal time-scale, photosynthetic gas exchange was measured at each time point (GFS-3000, Walz, Effeltrich, Germany). Measurements started 2h after the lights were turned on inside the growth chambers. A bundle of attached needles was oriented to form a flat plane and inserted in the leaf cuvette. CO_2_ concentration in the cuvette was set to 400 ppm, temperature was set to growth temperature (Table 1) and humidity was set at 60% RH. Net CO_2_ assimilation (*A*, in μmol CO_2_ m^–2^s^–1^) was measured at growth light intensity (1,400 μmol quanta m^–2^s^–1^) once steady-state assimilation was achieved. Immediately after the measurements, needles were detached from the seedling and measured for surface area using the WinSEEDLE software package (Regent Instruments Inc., Québec, Canada). The light-use efficiency of CO_2_ assimilation (LUE_A_, in mol CO_2_ mol^–1^ quanta) was calculated as:

$$LUE_{\text{A}}=\frac{Assimilation}{PPFD}$$ (12)

To assess variations in photosynthetic activity at a diurnal time-scale, light response curves were measured on days 0 and 12 in both treatments. A fully dark-adapted bundle of needles was exposed to a sequence of eight 10-min light steps of increasing actinic light intensity (0–2,000 μmol quanta m^–2^s^–1^). Measurement and cuvette conditions were identical to those used for assessing variation of photosynthetic activity at a seasonal time-scale (see above). At each light step, *A* was measured and LUE_A_ was calculated.

### Spectral reflectance measurements

Seasonal variations in PRI were assessed from leaf spectral reflectance measurements using a Unispec-SC spectrometer (UNI007, PP Systems, Haverhill, MA, USA) equipped with an internal tungsten halogen light source. The spectrometer was connected to a bifurcated fibre-optic (UNI400) and a leaf clip (UNI500) maintaining the fibre-optic on the needle surface at a fixed angle of 60° relative to needle axis (2mm diameter spot size). Leaf bidirectional reflectance was computed by dividing reflected irradiance by the radiance obtained from a white reflectance standard (Spectralon, Labsphere, North Sutton, NH, USA) taken immediately before each leaf measurement. Dark current instrument noise was subtracted from white standard and leaf radiance measurements. Reflectance was measured on needles of the topmost part of the leader shoot. Needles were in bundles of approximately 10–15 needles arranged in parallel to form a single layer flat plane. The integration time was set to 10ms and 40 scans were averaged for each measurement followed by interpolation of the ~3.3nm resolution output of the spectrometer to 1nm bandwidths using the software Multispec v. 5.1.0 (Purdue University, Indiana, USA). Finally, PRI was calculated according to Peñuelas *et al.* (1995):

$$PRI=\frac{R_{531}-R_{570}}{R_{531}+R_{570}}$$ (13)

where *R*_531_ and *R*_570_ represent leaf reflectance at 531 and 570nm, respectively. In order to compare seasonal PRI variation, pre-dawn PRI measurements were used to separate the effects of long-term and short-term pigment variations on PRI (Gamon and Berry, 2012).

To assess the effect of short-term xanthophyll pigment variations on PRI and infer the magnitude of diurnal PRI variation light response curves were measured on days 0 and 12. A bundle of needles was set up in the leaf clip and exposed to eight 10-min light steps of increasing actinic light intensity (0–2,000 μmol quanta m^–2^s^–1^) using the Unispec-SC internal light source. Three scans were averaged at each light step. To assess the range of diurnal PRI variation, ΔPRI was calculated as the difference between PRI of dark-adapted needles and PRI measured at 2,000 μmol quanta m^–2^s^–1^ (Gamon and Berry, 2012).

### Pigment analysis

Needle samples for pigment analysis were collected after at least 2h of exposure to growth light. The samples were immediately frozen in liquid nitrogen and stored at –80 °C. The needles were then ground to a fine powder in liquid nitrogen. Pigments were extracted in dim light conditions in 98% methanol buffered with 2% 0.5M ammonium acetate for 2h. The extracts were filtered through a 0.2 μm PTFE filter prior to high-performance liquid chromatography (HPLC) analysis. HPLC analysis was performed with an Agilent 1260 system (Agilent Technologies, Santa Clara, CA, USA) with a quaternary pump, autosampler set to 4 °C, column oven set to 25 °C, and a photodiode array detector. Pigments were detected at 450nm and 656nm wavelengths and separated using a reverse-phase C_30_ column (5 μm, 250×4.6mm; YMC Co. Ltd., Kyoto, Japan) protected by a 20×4.6mm guard column. Three solvents, i.e. A: 100% methanol, B: 100% methyl-*tert*-butyl-ether, and C: water buffered with 0.2% ammonium acetate, were used to run a gradient starting with 92% A, 5% B, and 3% C. During each run solvent A was gradually replaced by solvent B to a minimum of 5% A. Every run was followed by a 5min reconditioning phase with initial solvent concentrations. For calibration and peak detection, commercially available standards were obtained from Sigma Aldrich (St Louis, MO, USA) and DHI Lab products (Hørsholm, Denmark). Peak detection and pigment quantification was performed using ChemStation software (Agilent Technologies, Santa Clara, CA, USA).

Total chlorophylls (Chl) were determined as the sum of chlorophyll *a* and *b* concentration on a fresh weight basis (μmol g^–1^), as the water content of *P. strobus* needles varies less than 10% year-round (Verhoeven *et al.*, 2009). The ratio of Chl *a*/*b* was expressed in mol mol^–1^ and the concentration of carotenoid pigments was normalized to chlorophyll levels and was expressed in mmol carotenoids mol^–1^ Chl. Total carotenoids (Car) were expressed (in mmol mol^–1^ Chl) as the sum of violaxanthin, antheraxanthin, zeaxanthin, neoxanthin, lutein, α-carotene and β-carotene concentrations. Total xanthophyll cycle pigments (VAZ) were calculated as the sum of violaxanthin, antheraxanthin, and zeaxanthin. DEPS (in mol mol^–1^) was expressed as:

$$DEPS=\frac{0.5A+Z}{V+A+Z}$$ (14)

### Statistical analyses

The effects of treatments on individual parameters at each time point were estimated using mixed model analysis of variance (ANOVA). For all statistical analyses the data from two experiments run on two different years were pooled together. Year was included as a random effect in the mixed model to account for the two replicate experiments. The analysis was performed using the difflsmeans function in the lmerTest package, using R version 3.1.1 (R Core Team, 2014).

In order to evaluate the strength of the relationship between PRI and physiological parameters, *R* ^2^ values were obtained from linear regressions with log-transformed variables and the slope was considered significantly different from zero when *P* <0.05. Regressions were performed using GraphPad Prism 6 software version 6.05 (GraphPad Software, Inc., La Jolla, CA, USA).

## Results

### Seasonal variation in energy partitioning of PSII and PSI

**Fig. 1.**
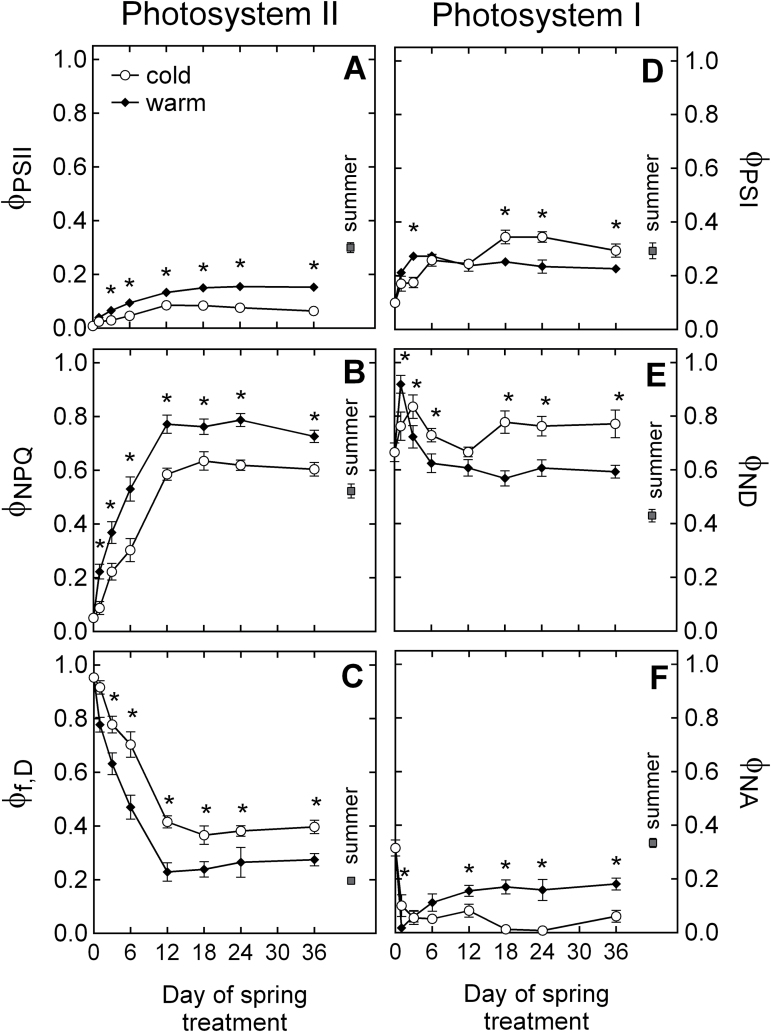
Time-course of energy partitioning characteristics of *P. strobus* needles exposed to cold and warm spring treatments. (A) Φ_PSII_, fraction of absorbed light at PSII used for photochemistry; (B) Φ_NPQ_, fraction of absorbed light at PSII dissipated via xanthophyll-regulated thermal dissipation; (C) Φ_f,D_, sum of fluorescence and constitutive thermal dissipation at PSII; (D) Φ_PSI_, effective photochemical quantum yield of PSI; (E) Φ_ND_, fraction of P700 oxidized due to a lack of electron donors; and (F) Φ_NA_, fraction of P700 that cannot be oxidized by a saturation pulse due to a lack of electron acceptors. Measurements were taken at 1400 μmol quanta m^–2^s^–1^ light intensity. Each data point represents *n*=10–11 seedlings ±SE and asterisks indicate significant differences (α <0.05) between treatments at a given time point.

Winter-acclimated seedlings (Wi) were assessed on day 0 of the experiment and then transferred to simulated spring growth conditions. Following transfer to spring conditions, a clear response of energy partitioning to longer photoperiod and warmer temperature was observed. In both spring treatments, the recovery of photosynthesis reached a steady state by day 12 of the experiment (Fig. 1A). By that time, the effective quantum yield of PSII (Φ_PSII_) was approximately twice as high in the warm spring treatment (Sp_W_) as in the cold spring treatment (Sp_C_). After photosynthetic recovery, 60% of absorbed energy in Sp_C_ was dissipated via xanthophyll-regulated NPQ (Φ_NPQ_) and 40% via fluorescence or dissipated constitutively (Φ_f,D_; Fig. 1B, C). In Sp_W_, however, the majority (75%) of NPQ was facilitated by Φ_NPQ_ (Fig. 1B). In Wi seedlings, more than 90% of all light energy absorbed was dissipated via Φ_f,D_, but after 18 d of exposure to spring conditions this value had declined to approximately 40% in Sp_C_ and 20% in Sp_W_ (Fig. 1C).

In addition to measurements of energy partitioning in PSII, energy conversion in PSI was assessed (Fig. 1D, E, F). Upon transfer to spring conditions, the effective quantum yield of PSI (Φ_PSI_) quickly recovered, with a faster rate in Sp_W_ compared with Sp_C_ (Fig. 1D). However, by day 18 of the experiment, Φ_PSI_ was higher in Sp_C_ and remained so until the end of the experiment. During that period, quantum yield in Sp_C_ was five times higher in PSI compared with PSII. By contrast, in Sp_W_ seedlings, the quantum yield of PSI was only 1.5 times higher than in PSII (Fig. 1A, D). In both spring treatments, the fraction of overall P700 oxidized due to a lack of electron donors (Φ_ND_) transiently increased on the first day of the experiment, and then gradually declined in the following days (Fig. 1E). Φ_ND_ stabilized to a steady state by day 18 of the experiment to values significantly higher in Sp_C_ than in Sp_W_. In both treatments, the fraction of overall P700 that cannot be oxidized by a saturation pulse due to a lack of electron acceptors (Φ_NA_) dropped on the first day of exposure to spring conditions (Fig. 1F). In Sp_C_, Φ_NA_ continued to decrease until the end of the experiment. By contrast, Φ_NA_ in Sp_W_ increased after day 1, until it reached a plateau by day 12 and then remained significantly higher than in Sp_C_ until the end of the experiment.

**Fig. 2.**
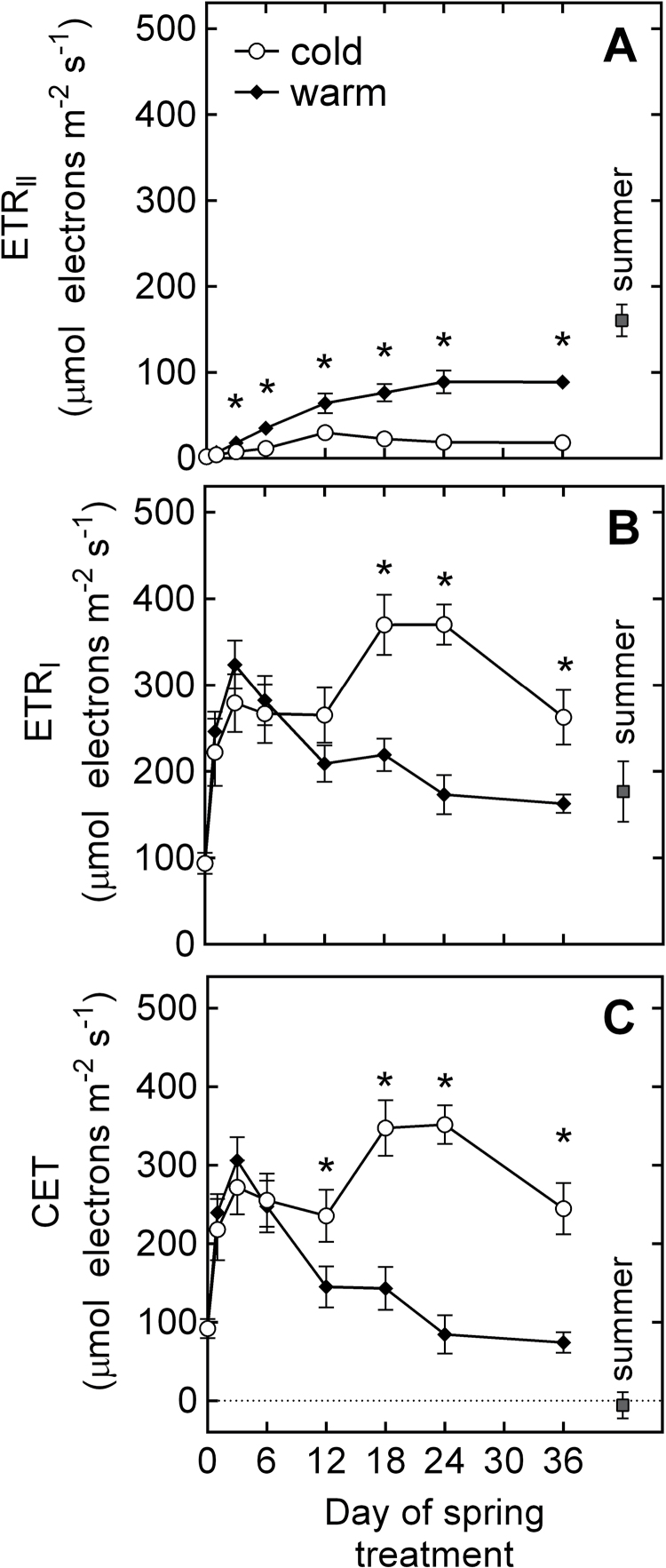
Time-course of (A) electron transport rate of PSII; (B) electron transport rate of PSI; and (C) cyclic electron transport rate of *P. strobus* needles exposed to cold and warm spring treatments. Measurements were taken at 1400 μmol quanta m^–2^s^–1^ light intensity. Each data point represents *n*=10–11 seedlings ±SE and asterisks indicate significant differences (α <0.05) between treatments at a given time point.

Acclimation to Wi conditions resulted in high *d*_I_ values (0.88±0.02) compared ith *d*_II_ (0.12±0.02). Exposure to both spring treatments resulted in a gradual increase in *d*_II_ and a decline in *d*_I_ as the experiment progressed, with generally higher *d*_I_ values in Sp_C_ than in Sp_W_ (Table 2). Throughout the experiment, the electron transport rate of PSI (ETR_I_) was higher than the electron transport rate of PSII (ETR_II_) in both spring treatments (Fig. 2A, B). The highest ETR_I_ values were observed in Sp_C_ between days 12 and 24 of the experiment. Cyclic electron transport (CET) was significantly higher in Sp_C_ than in Sp_W_ from day 12 of the experiment, with CET approximately 300% higher in Sp_C_ than in Sp_W_ during the second half of the experiment (Fig. 2C).

**Table 2.** Fraction of light absorbed by PSII and PSI (*d*_II_ and *d*_I_) and absorptance (α) of *P. strobus* needles exposed to winter, cold spring, warm spring, and summer treatments (*n*=11 seedlings ±SE)

Day	*d*_II_	*d*_I_	**α**
Winter	0.12±0.02	0.88±0.02	0.87±0.01
Cold spring			
1	0.12±0.01	0.88±0.01	0.86±0.01
3	0.19±0.03	0.81±0.03	0.88±0.01
6	0.15±0.02	0.85±0.02	0.88±0.01
12	0.25±0.02	0.75±0.02	0.87±0.01
18	0.20±0.02	0.80±0.02	0.88±0.01
24	0.18±0.02	0.82±0.02	0.87±0.01
36	0.22±0.02	0.78±0.02	0.86±0.01
Warm spring			
1	0.13±0.02	0.87±0.02	0.88±0.01
3	0.19±0.02	0.81±0.02	0.89±0.01
6	0.27±0.03	0.73±0.03	0.87±0.01
12	0.34±0.03	0.66±0.03	0.88±0.004
18	0.37±0.03	0.63±0.03	0.87±0.004
24	0.44±0.03	0.56±0.03	0.87±0.01
36	0.44±0.02	0.55±0.02	0.86±0.01
Summer	0.53±0.01	0.47±0.01	0.85±0.01

### Photosynthetic recovery and seasonal changes in PRI

**Fig. 3.**
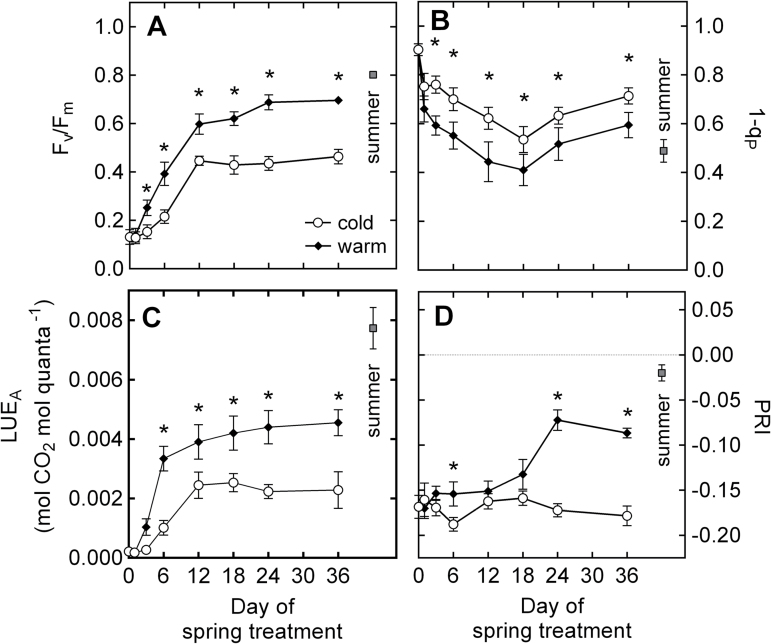
Time-course of photosynthetic recovery in *P. strobus* exposed to cold and warm spring treatments. (A) *F*_v_/*F*_m_, maximal quantum yield of PSII; (B) 1*–qP*, excitation pressure of PSII; (C) LUE_A_, light-use efficiency of photosynthetic CO_2_ assimilation; and (D) PRI. *F*_v_/*F*_m_ and PRI were measured on dark-acclimated needles and 1–*qP* and *LUE*_A_ were measured at 1400 μmol quanta m^–2^s^–1^ light intensity. Each data point represents *n*=10–11 seedlings ±SE and asterisks indicate significant differences (α <0.05) between treatments at a given time point.

Consistent with the pattern observed in energy partitioning (Fig. 1), a recovery of photosynthetic activity was observed over the 36 d of our experiment (Fig. 3). By the end of the experiment, the maximum quantum yield of PSII (*F*_v_/*F*_m_) had recovered from a value of 0.13 in Wi seedlings (day 0) to values of 0.46 in Sp_C_ and 0.70 in Sp_W_ (Fig. 3A). From day 3, *F*_v_/*F*_m_ was significantly higher in Sp_W_ than in Sp_C_. In both treatments, the excitation pressure of PSII (1–*qP*) declined from day 1 of the experiment until day 18, but then recovered considerably until day 36 of the experiment (Fig. 3B). Values of 1–*qP* were significantly higher in Sp_C_ during most of the experiment. In both treatments, the light-use efficiency of CO_2_ assimilation (LUE_A_) recovered quickly during the first 12 d of the spring treatment, with a faster rate of recovery in Sp_W_ than in Sp_C_ (Fig. 3C). LUE_A_ was consistently higher in Sp_W_ compared with Sp_C_ (Fig. 3C). Under winter conditions, a PRI value of –0.168 was recorded. In Sp_C_, PRI slightly declined until day 6 of the experiment and then increased again by day 12 and remained at winter-like values until the end of the experiment (Fig. 3D). In Sp_W_, PRI was also relatively stable for the first 12 d of the experiment, with most of its recovery occurring between days 12 and 24 of the experiment, and with final values of approximately –0.05. After day 24, PRI was significantly higher in Sp_W_ than in Sp_C_.

### Seasonal changes in foliar pigment content

**Fig. 4.**
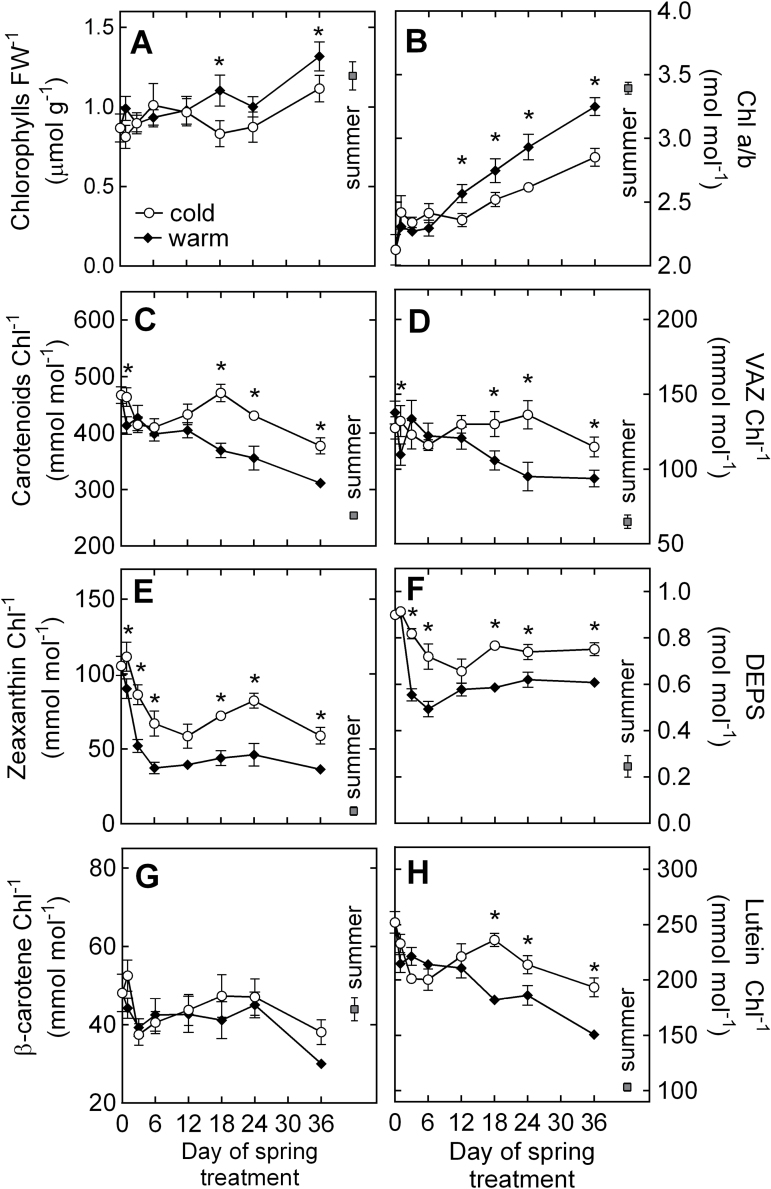
Time-course of photosynthetic pigment dynamics in *P. strobus* needles exposed to cold and warm spring treatments. (A) Chl FW^–1^; (B) ratio of Chl *a* to Chl *b*; (C) Car Chl^–1^; (D) VAZ Chl^–1^; (E) zeaxanthin Chl^–1^; (F) DEPS; (G) β-carotene Chl^–1^; and (H) lutein Chl^–1^. Each data point represents *n*=10–11 seedlings ±SE and asterisks indicate significant differences (α <0.05) between treatments at a given time point.

The transfer of seedlings from winter to spring conditions resulted in a clear response of photosynthetic pigment composition in both treatments (Fig. 4). Chlorophyll (Chl) FW^–1^ remained stable until day 12 of the experiment, but recovered to summer levels after day 18 (Fig. 4A). By the end of the experiment, the pool of Chl had increased by 30% in Sp_C_ and by 53% in Sp_W_. In both treatments, Chl *a*/*b* increased over the course of the experiment and, as of day 12, higher Chl *a*/*b* was observed in Sp_W_ than in Sp_C_ (Fig. 4B). By contrast, carotenoid (Car) Chl^–1^ peaked in Sp_C_ on day 18 of the experiment, and remained significantly higher in Sp_C_ than in Sp_W_ until day 36 (Fig. 4C). Xanthophyll cycle pigments (VAZ) Chl^–1^ remained fairly stable over the course of the experiment in Sp_C_ but declined in Sp_W_ seedlings after day 3 (Fig. 4D). The amount of zeaxanthin Chl^–1^ decreased over the first 6 d of the experiment in both treatments, increased again until day 24 in Sp_C_, and was significantly higher in Sp_C_ than in Sp_W_ for most of the experiment (Fig. 4E). By day 36, zeaxanthin had decreased by 44% in Sp_C_ and by 66% in Sp_W_ compared with Wi seedlings on day 0. A similar trend was observed for the de-epoxidation status of the xanthophyll cycle (DEPS; Fig. 4F), which was higher in Sp_C_ compared with Sp_W_ throughout most of the experiment. For instance, by day 36 DEPS was 0.75 and 0.61mol mol^–1^ in Sp_C_ and Sp_W_, respectively. In both treatments, the amount of β-carotene Chl^–1^ also declined throughout the experiment. Most of the β-carotene Chl^–1^ was lost after day 24 of the experiment, with 21% of the winter β-carotene pool lost in Sp_C_, and 38% lost in Sp_W_ (Fig. 4G). At any time during the experiment, the amount of β-carotene did not significantly differ between treatments. Lutein Chl^–1^ in Sp_C_ initially declined on the first days of the experiment, and then increased until day 18, before it decreased again until the end of the experiment. In Sp_W_, lutein Chl^–1^ decreased consistently throughout the experiment (Fig. 4H). From day 18, significantly higher levels of lutein were observed in Sp_C_ compared with Sp_W_.

### Variation in the relationships between PRI and physiological parameters during the winter–spring transition

**Fig. 5.**
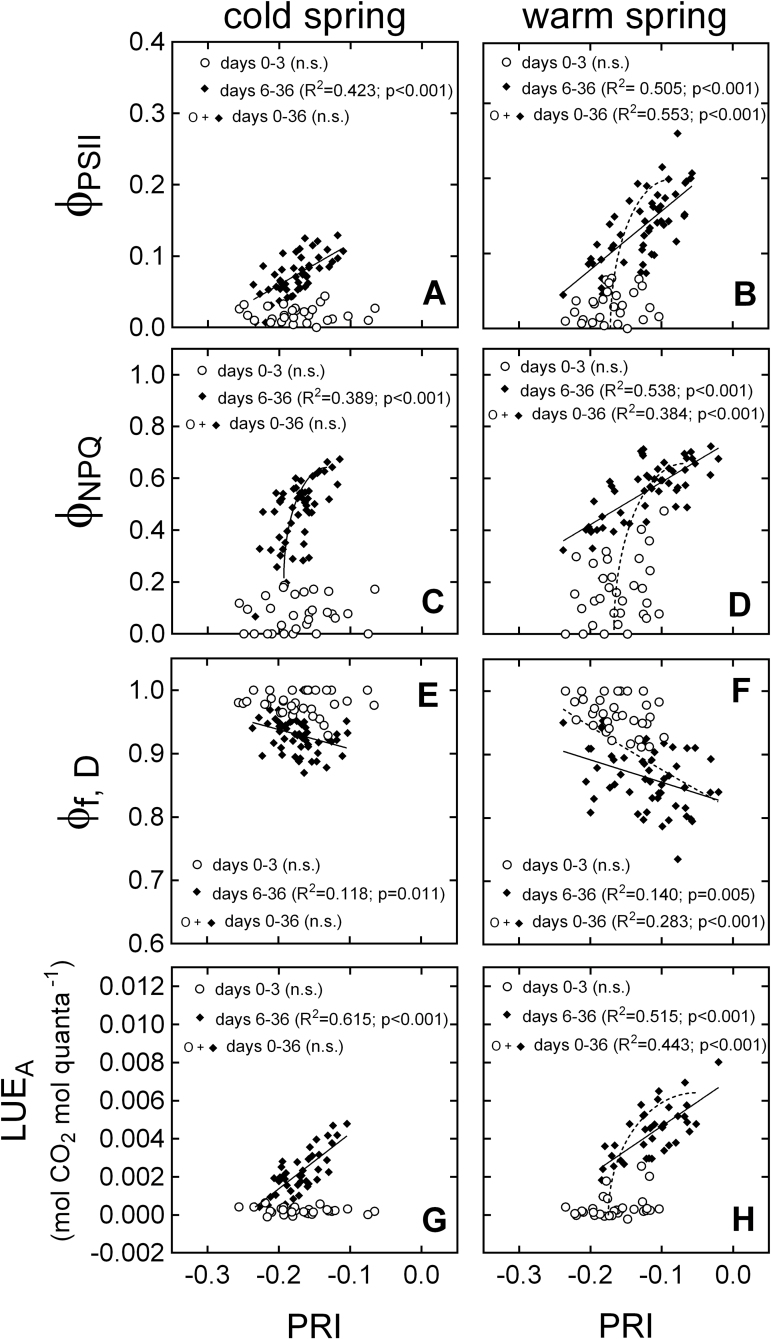
Relationship between PRI and (A, B) Φ_PSII_, fraction of absorbed light used for photochemistry; (C, D) Φ_NPQ_, fraction of absorbed light quenched via xanthophyll-regulated thermal dissipation; (E, F) Φ_f, D_, sum of fluorescence and constitutive thermal dissipation; (G, H) LUE_A_, light-use efficiency of CO_2_ assimilation during the photosynthetic recovery of *P. strobus* exposed to cold and warm spring treatments. *R* ^2^ values indicate goodness of fit for linear, logarithmic or exponential relationships during days 0–3 of the experiment (circles), days 6–36 (diamonds, full line) or the entire experiment (circles and diamonds, dashed line). The left and right panels present data from the cold and warm treatments, respectively.

**Fig. 6.**
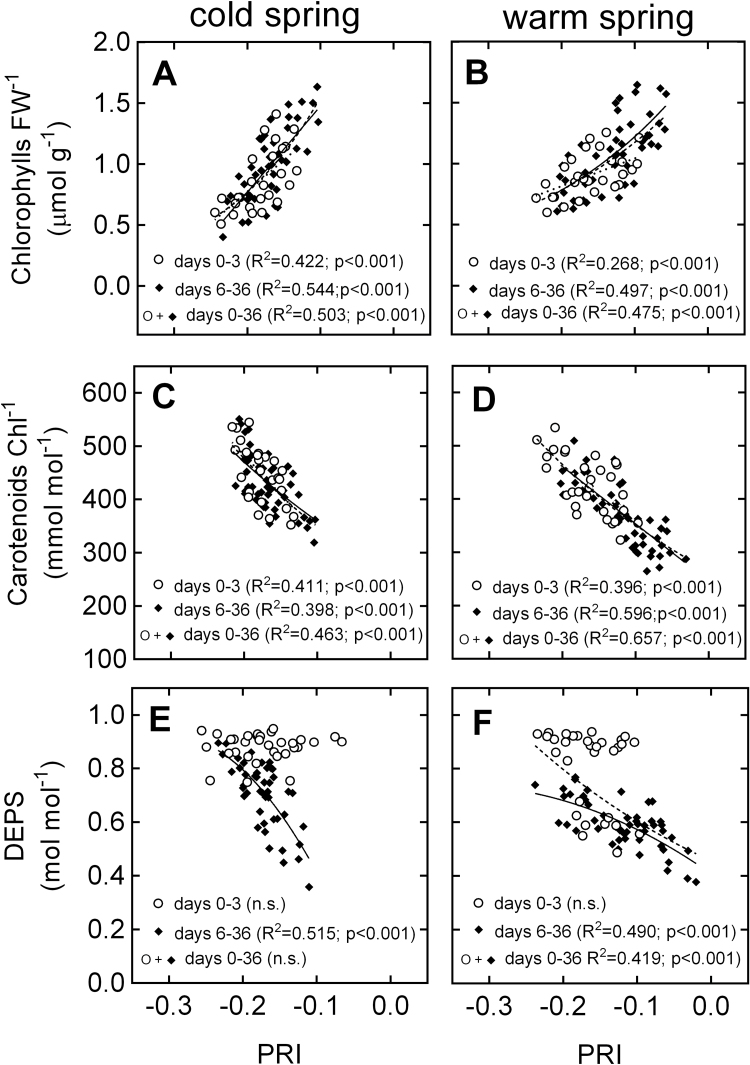
Relationship between PRI and (A, B) Chl FW^–1^; (C, D) Car Chl^–1^; and (E, F) DEPS during the photosynthetic recovery of *P. strobus* exposed to cold and warm spring treatments. *R* ^2^ values indicate goodness of fit for exponential or linear relationships for days 0–3 of the experiment (circles, dotted line), days 6–36 (diamonds, full line) or the entire experiment (circles and diamonds, dashed line). The left and right panels present data from the cold and warm treatments, respectively.

The relationship between PRI and energy partitioning varied depending on exposure time to the treatments (Fig. 5). For all energy partitioning parameters, no relationship with PRI was observed during days 0–3 of the experiment, but significant relationships for days 6–36 and/or the entire experiment (Fig. 5). Similar trends were observed for DEPS, where samples from days 0–3 were clearly separated from samples taken between days 6–36 (Fig. 6E, F). By contrast, relationships between PRI and Chl FW^–1^ or Car Chl^–1^ were significant for all time periods (Fig. 6A–D).

### Response of photosynthesis to short-term variations in light intensity of seedlings acclimated to winter, spring or summer conditions

**Fig. 7.**
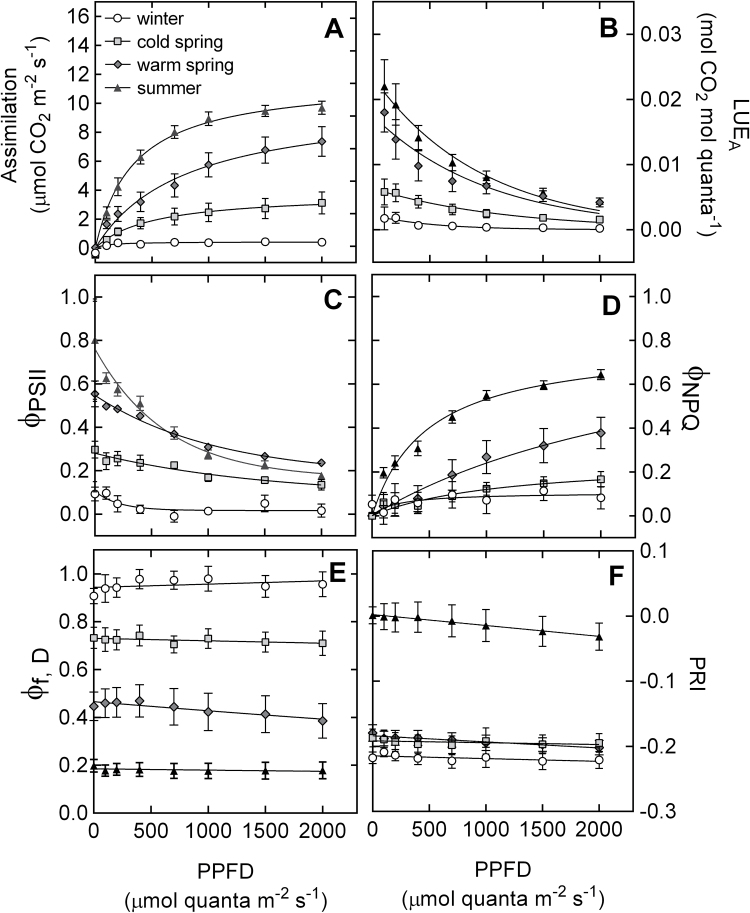
The response to light of *P. strobus* needles acclimated to winter, cold spring, warm spring or summer conditions for (A) photosynthetic CO_2_ assimilation; (B) LUE_A_, light-use efficiency of CO_2_ assimilation; (C) Φ_PSII_, fraction of absorbed light used for photochemistry, (D) Φ_NPQ_, fraction of light quenched via xanthophyll-regulated thermal dissipation; (E) Φ_f,D_, sum of fluorescence and constitutive thermal dissipation; and (F) PRI. Each data point represents *n*=6–8 seedlings ±SE.

After 12 d of acclimation to simulated spring conditions, the seedlings exhibited different responses to increasing light intensity compared with winter conditions (Fig. 7). Assimilation increased from almost 0 μmol CO_2_ m^–2^s^–1^ in Wi seedlings to approximately 3 μmol CO_2_ m^–2^s^–1^ in Sp_C_ and 7 μmol CO_2_ m^–2^s^–1^ in Sp_W_ at 2,000 μmol quanta m^–2^s^–1^ light intensity (Fig. 7A). Assimilation in Sp_C_ and Sp_W_ had recovered 31% and 76% of the capacity observed in Su at 2,000 μmol quanta m^–2^s^–1^ light intensity. The higher maximum rate of assimilation in spring seedlings was accompanied by lower light compensation points compared with Wi (122.7±42.9 μmol quanta m^–2^s^–1^), with 80.7±23.1 μmol quanta m^–2^s^–1^ in Sp_C_ and 14.2±8.6 μmol quanta m^–2^s^–1^ in Sp_W_. Compared with winter, LUE_A_ was slightly higher in Sp_C_, while LUE_A_ in Sp_W_ was only slightly lower than Su values (Fig. 7B). Assessing the changes in energy partitioning via chlorophyll-fluorescence revealed a different pattern. The short-term light response of Φ_PSII_ in Sp_W_ was very close to Su values, but considerably higher Φ_NPQ_ and lower Φ_f,D_ values were observed over the full range of light intensities in Sp_W_ (Fig. 7C, D, E). By contrast, Sp_C_ seedlings showed Φ_NPQ_ values similar to Wi, but considerably higher Φ_PSII_ and lower Φ_f,D_. PRI was still very close to winter values in both spring treatments, which had recovered approximately 18% of their Su values (Fig. 7F). The range of diurnal PRI variation differed between treatments, with a ΔPRI of –0.033 in Su, a ΔPRI of –0.008 in Sp_C_, a ΔPRI of –0.022 in Sp_W_, and a ΔPRI of –0.0032 in Wi.

## Discussion

### Conifers undergo a reorganization of components of the chloroplast during winter stress

The winter conditions simulated in our experiment induced the complete down-regulation of photosynthesis in pine seedlings (Fig. 3C) including a 30% reduction of the chlorophyll that was present in summer-acclimated needles (Fig. 4A). Light absorption by chlorophylls remaining in the winter-acclimated needles resulted in high excitation pressure of PSII, as indicated by 1–*qP* values close to 1 (Fig. 3B). Low *F*_v_/*F*_m_ values observed under winter conditions indicated that the chlorophyll pigments were retained in a quenched, photoprotected state (Ottander *et al.*, 1995; Ensminger *et al.*, 2004). Under winter conditions, approximately 90% of all energy absorbed by pine needles was dissipated thermally via sustained NPQ (Fig. 1C) as reported in winter-acclimated *P. strobus* trees (Verhoeven, 2013). This sustained capacity for NPQ was facilitated by xanthophyll cycle pigments maintained in a highly de-epoxidized state compared with the summer seedlings (Fig. 4D, F). Accumulation of xanthophylls was also accompanied by larger amounts of lutein and β-carotene, reflecting an increased capacity for triplet Chl quenching (Fig. 4G, H) that has been observed in several other overwintering conifer species (Adams and Demmig-Adams, 1994; Ottander *et al.*, 1995, Sveshnikov *et al.*, 2006). This winter state concurred with highly negative PRI values of approximately –0.2 (Fig. 3D), which is comparable to PRI values reported in winter down-regulated pine species (Wong and Gamon, 2015*a*, *b*).

Although the functionality of both photosystems was impaired under winter conditions, PSI was less affected than PSII (Table 2; Fig. 1, 2). While PSII electron transport was suppressed almost completely (Fig. 2A), approximately 50% of the capacity observed in summer needles was preserved in PSI (Fig. 2B). This is in accordance with Ivanov *et al.* (2001) who observed that PSI has a higher level of resistance to winter stress compared with PSII. The repression of linear electron transport downstream of PSII was accompanied by marked donor-side limitation of PSI, as indicated by high Φ_ND_ values (Fig. 1E). Interestingly, Φ_NA_ in winter-acclimated needles was the same as that observed during summer, suggesting a retention of the pool size of PSI electron acceptors during winter-acclimation, possibly as a strategy to facilitate rapid recovery of carbon fixation in the early spring. The observed imbalance between electron transport at PSII and PSI points to enhanced cyclic electron transport around PSI (Johnson, 2011). This indicates that, in winter-acclimated seedlings, electron flow around PSI plays a considerable role in the removal of excess light. Oxidized P700 can efficiently quench chlorophyll fluorescence (Öquist and Hüner, 2003); thus, it is concluded that PSI was probably an important quencher of absorbed light in our winter-acclimated seedlings.

It is important to note that, in this study, needle absorptance (α) was calculated with the assumption that needle transmittance is zero. It was shown recently that a small proportion of light (<5% of the visible spectrum) can be transmitted through conifer needles (Lukeš *et al.*, 2013). The absorptance values presented in Table 2 might, therefore, slightly overestimate the true α, which can potentially result in a minor overestimation of ETR. However, the error is minimal and equally affects ETR_II_ and ETR_I_ and the overall trends observed in our data are not affected. It is recommended that future studies include measurements of reflectance and transmittance to facilitate the estimation of leaf absorptance and the calculation of α and ETR.

### Different rates of recovery cause decoupling of PRI and LUE during the early stages of spring

Immediately after transfer to our spring treatments, a decrease of the excitation pressure of PSII was observed (1–*qP*, Fig. 3B), reflecting the role of air temperature in restoring the redox state of the chloroplast (Demmig-Adams and Adams, 2006; Ensminger *et al.*, 2008). In both spring treatments, the maximum quantum yield of PSII recovered rapidly, indicating a fast reorganization of the photosynthetic apparatus under warmer conditions. A considerable recovery of assimilation was also observed within the first days of exposure to spring treatments (Fig. 3C). A concomitant decrease in zeaxanthin pools and a decrease in the de-epoxidation state of the xanthophyll cycle (Fig. 4E, F) were also observed, both indicating a decreased requirement for photoprotection. In *P. sylvestris*, Ensminger *et al.* (2008) also reported the rapid recovery of photosynthetic capacity, along with the rapid relaxation of DEPS, during the first few days of exposure to a simulated spring treatment. In our experiment, the quick decline in zeaxanthin Chl^–1^ and DEPS occurred concomitantly with the transition from sustained quenching to dynamic quenching mediated by xanthophyll cycle pigments (Fig. 1B, C).

Interestingly, under warm spring conditions, it took more than 18 d before PRI recovered from winter stress while, under cold spring conditions, no PRI recovery was observed. Similarly, levels of Chl FW^–1^ and Car Chl^–1^ did not recover from winter stress until after day 12 (Fig. 4A, C). In the field, boreal spring conditions are characterized by large day-to-day variations in air temperature, and photosynthesis recovers quickly and opportunistically with increasing air temperature. Conversely, photosynthesis can rapidly revert back to a down-regulated state with the occurrence of cold episodes (Ensminger *et al.*, 2004, 2008). Maintaining large pools of carotenoids in needles after photosynthetic recovery reflects a strategy that allows for rapid photoprotection during sudden low temperature episodes. On the other hand, the delay in Chl synthesis during the early stages of photosynthetic recovery prevents the absorption of excess light and photo-oxidative stress. This is supported by the fact that the timing of Car down-regulation and Chl up-regulation coincided with the occurrence of the maximum rates of photosynthesis after day 12 of the experiment (Figs 3, 4). A mismatch in the timing of the spring recovery was also reported by Wong and Gamon (2015b). They observed that the recovery of electron transport, LUE, and the epoxidation status of the xanthophyll cycle (EPS) occurred approximately 2–3 weeks earlier than the recovery of PRI in *P. ponderosa* and *P. contorta* seedlings exposed to natural spring conditions. These differences in the timing of recoveries indicate that, during the early stages of photosynthetic recovery, xanthophyll cycle dynamics are not the main factor controlling PRI. PRI therefore insufficiently detects variations in LUE during that time.

### At a seasonal time-scale, PRI is mostly controlled by carotenoid and chlorophyll pool sizes

Previous studies on boreal pine species have reported a strong contribution of pigment pools on PRI. For instance, Porcar-Castell *et al.* (2012) reported that variations in the pool size of xanthophyll cycle pigments over the year control the dynamics of PRI in *P. sylvestris*, while Wong and Gamon (2015*a*, *b*) reported a combined effect of all carotenoid pigments in *P. ponderosa* and *P. contorta*. Our experiment revealed that PRI and DEPS did not correlate well during the early stages of spring recovery (days 0–3), when NPQ was predominantly facilitated via the sustained quenching mode. By contrast, PRI correlated consistently with Chl and Car pigment pool sizes throughout the spring transition, regardless of the degree of photosynthetic recovery (Fig. 6A–D). Furthermore, Chl and Car were responsible for a large proportion of the PRI variation observed. The range of PRI variation between winter and summer seedlings was 0.22 while the largest range of PRI variation observed at a diurnal scale was only 0.033 (Fig. 7F). Accordingly, the magnitude of variation in PRI due to long-term pigment pool adjustments was more than six times higher than the magnitude caused by short-term changes in xanthophyll cycle pigments. This ratio suggests that, on an annual scale, the PRI signal reflects the small contribution of xanthophyll cycle dynamics superimposed on the much larger effects of seasonal pigment adjustments. Thus, the adjustments in pigment pools conceal the diurnal variations in PRI, especially during the winter–spring transition from sustained NPQ to energy-dependent NPQ.

### Alternative energy sinks contribute to the early spring discrepancy in the PRI–LUE relationship

During the spring transition from sustained to reversible zeaxanthin-dependent NPQ, adjustments in NPQ may occur independently of xanthophyll pigment conversions and decouple PRI from NPQ. This was observed during the entire duration of the spring simulation, where sustained quenching was retained to some extent in both the cold and warm treatments and particularly during the first days of exposure to spring conditions. What causes the delay of the release of sustained quenching? Sustained NPQ or the thermal dissipation of excess energy is achieved through structural reorganization of PSII. These changes include the aggregation of LHCII as a major energy dissipation pathway (Horton *et al.*, 1991, 2005; Ottander *et al.*, 1995). Unlike the xanthophyll-regulated LHCII aggregation associated with energy-dependent quenching (Horton *et al.*, 2005; Ruban *et al.*, 2012), this cold-induced reorganization reflects a zeaxanthin-independent quenching mechanism which is not detected by PRI. In addition to LHCII aggregation, another zeaxanthin-independent electron sink that remains undetected by PRI is the diversion of excess energy to PSI-driven cyclic electron transport. In *P. strobus* seedlings, the recovery of PSII function was generally slowed down and impaired in the cold spring treatment. PSII electron transport in the cold spring treatment recovered to values approximately half of that observed in the warm spring treatment and at a slower recovery rate (Fig. 2A). Interestingly, PSI electron transport in the cold spring treatment greatly surpassed the amount recorded in the warm treatment during the second half of the experiment (Fig. 2B). Ivanov *et al.* (2001) suggested that PSI photochemistry in early spring supplies the ATP required to maintain the integrity of chloroplasts while supporting recovery from winter stress. In this experiment, enhanced PSI-driven electron transport coincided with a high excitation pressure of PSII (Figs 2C, 3B). High light and low temperature in our cold spring treatment imposed increased excitation pressure and probably photodamage to the D1 protein of PSII. Because *de novo* synthesis of the D1 protein requires a large amount of ATP (Murata *et al.*, 2007), cyclic electron flow was proposed to be an essential component in the recovery of PSII under conditions of impaired linear electron transport (Huang *et al.*, 2010). This may indicate that, in the cold spring treatment, a larger fraction of absorbed energy was invested in ATP production via PSI-driven electron transport, rather than being dissipated as heat via the xanthophyll cycle as was the case in the warm spring treatment.

Under conditions of cold stress, both PSII and PSI may be important non-radiative quenchers through charge recombination events (Lunde *et al.*, 2000; Öquist and Hüner, 2003). In addition, Savitch *et al.* (2010) suggested that PTOX-mediated electron transport to oxygen is a major electron sink during winter. However, such measurements were outside the scope of this experiment, but these energy sinks probably contribute to NPQ during the winter–spring transition without apparent effect on PRI. This early spring decoupling of PRI and LUE seems to result in the overestimation of LUE (Fig. 5A, B), as reported by Porcar-Castell *et al.* (2012) in *P. sylvestris* during early spring, when foliage was down-regulated due to severe cold stress.

## Conclusions

Our results demonstrate an early spring decoupling in the PRI–LUE relationship caused by differences between the timing of the recovery of photosynthesis and the timing of carotenoid and chlorophyll pigment pool size adjustments which are the main controlling factors of PRI during spring. It is also demonstrated that zeaxanthin-independent NPQ mechanisms undetected by PRI further contributed to this decoupling. One of the main mechanisms contributing to the decoupling of the PRI–LUE relationship probably involves PSI-driven electron transport, which appears to be a significant energy sink during the entire spring transition, particularly in needles exposed to a combination of high light and cold temperatures.

Future studies should also aim to validate the mechanisms identified here on mature trees and in natural systems where additional causes of PRI variation, such as illumination angle or canopy structure, are likely to impose additional complexity to the signal detected from leaf reflectance measurements.

## Abbreviations:

A: CO_2_ assimilation (in μmol CO_2_ m^–2^ s^–1^)
ATP: adenosine triphosphate
α: needle absorptance
Car: total carotenoids (in mmol mol^–1^ Chl)
CET: cyclic electron transport rate (in μmol electrons m^–2^s^–1^)
Chl: total chlorophylls (in μmol g^–1^ fresh weight)
DEPS: de-epoxidation status of the xanthophyll cycle (in mol mol^–1^)
ETR_II_: electron transport rate of PSII (in μmol electrons m^–2^s^–1^)
*d*_II_: fraction of light directed to PSII
*d*_I_: fraction of light directed to PSI
ETR_I_: electron transport rate of PSI (in μmol electrons m^–2^s^–1^)
*F*_*o*_: minimal fluorescence of light-adapted needles
*F*_m_: maximum fluorescence of dark-adapted needles
*F*_s_: yield of fluorescence of dark-adapted needles
*F*_v_/*F*_m_: maximum quantum yield of PSII
$F_{\text{m}}^{\prime },$: maximum fluorescence of light-adapted needles
$F_{\text{o}}^{\prime },$: minimal fluorescence of light-adapted needles
LUE: light-use efficiency of photosynthesis
LUE_A_: light-use efficiency of CO_2_ assimilation (in mol CO_2_ mol^–1^ quanta)
NPQ: non-photochemical quenching
P: P700 signal
*P*_m_: difference between fully reduced and fully oxidized P700
$P_{\text{m}}^{\prime },$: maximum change of the P700 signal upon application of SP
*P*_o_: minimal P700 signal
PPFD: photosynthetic photon flux density (in μmol quanta m^–2^s^–1^)
PRI: photochemical reflectance index
ΔPRI: difference between PRI of dark-adapted needles and PRI measured at 2,000 μmol quanta m^–2^s^–1^
PS: photosystem
PTOX: plastid terminal oxidase
SP: saturating pulse
Sp_C_: cold spring treatment seedlings
Sp_W_: warm spring treatment seedlings
Su: summer-acclimated seedlings
Φ_f,D_: energy quenched by fluorescence and dissipated constitutively
Φ_NA_: fraction of overall P700 that cannot be oxidized by a saturation pulse due to a lack of electron acceptors
Φ_ND_: fraction of overall P700 oxidized due to a lack of electron donors
Φ_NPQ_: proportion of light absorbed by PSII antenna and thermally dissipated via xanthophyll-regulated NPQ
Φ_PSI_: effective quantum yield of PSI
Φ_PSII_: effective quantum yield of PSII of light-adapted needles
1–*qP*: excitation pressure of PSII
VAZ: total xanthophyll cycle pigments (in mmol mol^–1^ Chl)
Wi: winter-acclimated seedlings.
